# Acceleration of Early-Photon Fluorescence Molecular Tomography with Graphics Processing Units

**DOI:** 10.1155/2013/297291

**Published:** 2013-03-31

**Authors:** Xin Wang, Bin Zhang, Xu Cao, Fei Liu, Jianwen Luo, Jing Bai

**Affiliations:** ^1^Department of Biomedical Engineering, Tsinghua University, Beijing 100084, China; ^2^Center for Biomedical Imaging Research, Tsinghua University, Beijing 100084, China

## Abstract

Fluorescence molecular tomography (FMT) with early-photons can improve the spatial resolution and fidelity of the reconstructed results. However, its computing scale is always large which limits its applications. In this paper, we introduced an acceleration strategy for the early-photon FMT with graphics processing units (GPUs). According to the procedure, the whole solution of FMT was divided into several modules and the time consumption for each module is studied. In this strategy, two most time consuming modules (*G_d_* and *W* modules) were accelerated with GPU, respectively, while the other modules remained coded in the Matlab. Several simulation studies with a heterogeneous digital mouse atlas were performed to confirm the performance of the acceleration strategy. The results confirmed the feasibility of the strategy and showed that the processing speed was improved significantly.

## 1. Introduction

Fluorescence molecular tomography (FMT) is a promising imaging technique for small animals that allows visualization of 3D distributions of fluorescent biomarkers *in vivo* [1, 2]. However, significant challenges remain in FMT because the high degree of light scatter in biological tissues results in an ill-posed image reconstruction problem and consequently reduces the spatial resolution [3]. Considering this point, time-gated technique is proposed, which only utilizes “early-arriving” photons that experience few scattering events so as to reduce the large amount of diffusion photons. To date, a number of groups have validated that with time-gated detection technique, the spatial resolution and fidelity of the reconstructed results can be improved [3–5].

For the reconstruction of FMT using early photons, there are several feasible algorithms, such as the filtered back-projection method, schemes based on the time-resolved diffusion equation (DE), the time-resolved telegraph equation (TE), and the second-order cumulant approximation of the radiative transport equation (RTE), [3–7]. Among them, the method based on time-resolved DE is the most popular utilized for simplicity. However, compared with continuous wave FMT (CW-FMT), time-domain FMT (TD-FMT) will cost more time because of the time scale. Generally, solving TD-FMT will cost tens of minutes to hours and there are no efficient schemes for its acceleration at present.

Fortunately, the high-speed development of graphics processing unit (GPU) technology provides direction to the acceleration of TD-FMT solution. The highly parallel structure of GPU makes it more effective than central processing unit (CPU) for a range of algorithms on parallelizable floating point operations. However, programming on GPU had been difficult until the compute unified device architecture (CUDA) was proposed in 2006 [8]. CUDA comes with a software environment that allows developers to use C as a high-level programming language. Utilizing CUDA-enabled GPU, parallel acceleration algorithms has been studied in the field of fluorescence tomography. Fang and Boas reported a parallel Monte Carlo algorithm accelerated by GPU for modeling time-resolved photon migration in arbitrary 3D turbid media [9]. Zhang et al. implemented acceleration of adaptive finite element framework for bioluminescence tomography with CUBLAS and CULA libraries [10]. However, to date, CUDA-enabled GPU technology has not been utilized to solve TD-FMT.

In this paper, we introduced an acceleration strategy for the early-photon FMT. The time consumption of each module was studied to confirm the necessity of GPU acceleration. In the strategy, two most time consuming modules (*G*_*d*_ and *W* modules) were accelerated with CUDA language, respectively, and the other modules were coded in the Matlab. Several simulations with a heterogeneous digital mouse atlas were performed to evaluate the performance of the acceleration strategy.

The paper is organized as follows. In Section 2, the forward and inverse models based on TD-FMT are illustrated in detail. Numerical simulations with fluorescence targets embedded in a 3D mouse model are carried out. In Section 3, simulation results are shown and analyzed. Finally, we discuss the results and conclusion in Section 4.

## 2. Materials and Methods

### 2.1. Time-Domain Diffusion Equation and Finite Element Method

The radiative transfer equation (RTE) is considered as the most accurate model for describing the process of photon propagation in biological tissues. However, because RTE is computationally expensive, the diffusion approximation of RTE is commonly used. Thus, photon propagation for FMT can be modeled with the coupled time-domain DEs as follows [7]:

(1) 1c∂Φx(r,t)∂t+μa(r)Φx(r,t)−∇·[D(r)∇Φx(r,t)] =δ(r−rs,t)1c∂Φm(r,t)∂t+μa(r)Φm(r,t)−∇·[D(r)∇Φm(r,t)] =η(r)τ[Φx(r,t)∗E(t)],

where Φ_*x*,*m*_(*r*, *t*) denotes the photon density for excitation and fluorescence light, respectively. *δ*(*r* − *r*_*s*_, *t*) provides the impulse light source. *μ*_*a*_ is the absorption coefficient and *μ*_*s*_′ is the reduced scattering coefficient. *D*(*r*) is the diffusion coefficient defined by *D*(*r*) = 1/(3(*μ*_a_(*r*) + *μ*_*s*_′  (*r*))). As the excitation and emission wavelength are close to each other, the optical properties are assumed to be identical at both excitation and emission wavelengths for simplification. The fluorescent targets are described by fluorescent distribution *η*(*r*) and lifetime *τ*.  *E*(*t*) = exp⁡(−*t*/*τ*) is the lifetime function. ∗ is the temporal convolution operator. *c* is the speed of light.

To solve these equations, Robin boundary conditions are implemented on the boundary ∂*Ω* of the region *Ω* [7]:

(2) $$\begin{array}{c} 2qD\left(r\right)\frac{\partial \Phi \left(r\right)}{\partial \vec{n}}+\Phi \left(r\right)=0, \end{array}$$

where $\vec{n}$ denotes the outward normal vector of the boundary. The coefficient *q* takes into account the refractive index mismatch between both media.

Based on the first-order Born approximation, the fluorescence signal Φ_*m*_(*r*_*sd*_, *t*) measured at a detector point *r*_*d*_ for an impulsive excitation at source position *r*_*s*_ at time *t* can be written as

(3) $$\begin{array}{c} \Phi_{m}\left(r_{sd},t\right)=\int_{\Omega }W\left(r_{sd},r,t\right)\cdot \eta \left(r\right)dr^{3}. \end{array}$$

The weight matrix *W*(*r*_*sd*_, *r*, *t*) is described as

(4) $$\begin{array}{c} W\left(r_{sd},r,t\right)=G\left(r_{s},r,t\right)*E\left(t\right)*G\left(r,r_{d},t\right), \end{array}$$

where *G*(*r*_*s*_, *r*, *t*) and *G*(*r*, *r*_*d*_, *t*) are Green's functions of excitation and emission (*G*_*s*_ and *G*_*d*_ in short). In addition, for an isotropic impulse source, *G* is equal to Φ.

In order to reduce the influence of heterogeneity, the normalized Born approximation [11] is employed as follows:

(5) $$\begin{array}{c} \Phi^{\text{nB}}\left(r_{sd},t\right)=\frac{\Phi_{m}\left(r_{sd},t\right)}{\Phi_{x}\left(r_{sd},t\right)}=\int_{\Omega }W^{\text{nB}}\left(r_{sd},r,t\right)\cdot \eta \left(r\right)dr^{3}, \end{array}$$

where *W* ^nB^ is the normalized Born approximation of *W*.

By utilizing the standard Galerkin-FEM method, the object is discretized into *N* mesh nodes and the time is approximated with a sequence of time points with a time interval Δ*t*. Then, Green's functions can be derived:

(6) (K+CΔt)Gv(n,k+1)=CΔtGv(n,k)+Sv(n,k)Gv(n,−1)=Gv(n,0)=0,

where *K* and *C* are matrices of *N* × *N* with the same expression as given in [7, 12, 13]

(7) Kij=∫Ω[D(r)·∇ui(r)·∇uj(r)+μa(r)ui(r)uj(r)]dΩ+12q∫∂Ωui(r)uj(r)d(∂Ω)Cij=1c∫Ωui(r)uj(r)dΩ

but *S*_*v*_(*n*, *k*) differs in form:

(8) $$\begin{array}{c} S_{v}\left(n,k\right)=\{\begin{array}{cc} \int_{\Omega }\delta \left(n,k\right)u_{i}\left(r\right)d\Omega & v=x\\ C\frac{c\eta \left(n\right)}{\tau }G_{x}\left(n,k\right)*E\left(k\right) & v=m. \end{array} \end{array}$$

At last, (3) is converted into the following matrix-form equation:

(9) $$\begin{array}{c} \Phi^{\text{nB}}=W^{\text{nB}}\cdot \eta . \end{array}$$

Then the unknown fluorescence distribution *η* at different time-gates is obtained by solving the linear equation (9) using algebraic reconstruction technique (ART) with nonnegative constraints.

### 2.2. GPU Acceleration Strategy

#### 2.2.1. The Flow Chart of the Acceleration Strategy

**Figure 1:**
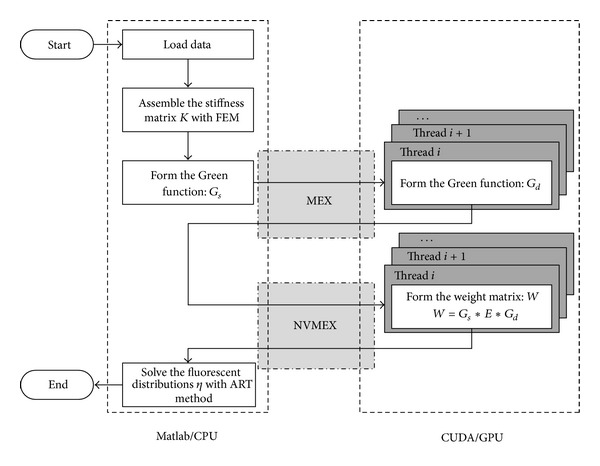
The execution flow chart of the whole acceleration strategy.

For the whole procedure, there is a large amount of matrix operations which are suitable for parallel accelerations by GPU. However, besides the matrix operations, there are still some other operations such as parameter configurations and mesh discretization, which are not suitable for the GPU acceleration. Therefore, the rest parts will be implemented in Matlab for programming flexibility. The execution flow chart of the whole algorithm is shown in Figure 1. The main program which contains the parts unnecessary to be accelerated is executed in Matlab. The parts of *G*_*d*_ and weight matrix acceleration, which need to be accelerated by GPU, are coded into subroutines so as to be called by the Matlab program. For the *G*_*d*_ acceleration, because CUBLAS library is used for the subroutine which can be recognized by the C compiler, “Matlab executable” (MEX) technology is available for the interface between the Matlab program and the *G*_*d*_ acceleration. As to the weight matrix acceleration, CUDA language is used in the subroutine and thus NVMEX technology is utilized as the interface. Details about the acceleration algorithms and the NVMEX technology are illustrated in the next subsections.

#### 2.2.2. *G*_*d*_ Acceleration

In the calculation procedure, the module to solve *G*_*d*_ is time consuming because matrix inversion should be performed for each detector at each time node. Although the method to solve *G*_*s*_ is similar to that of *G*_*d*_, the number of light sources is much smaller than the number of detectors. As a result, the time consumption of *G*_*s*_ is very little that it is unnecessary to be accelerated. The Matrix inversion of large size is computationally complex and there are no effective methods for this problem. Fortunately, the matrices that need to be inversed for each detection point and each time node are the same. Therefore, the inversion of the matrix can be calculated in advance and thus the inversion operations can be converted into multiplication operations, which can be accelerated by GPU more effectively.

NVIDIA has provided a CUBLAS library on top of the CUDA driver for the developers to do some basic linear algebra operations. CUBLAS is an implementation of basic linear algebra subprograms (BLAS) and the “CU” stands for CUDA [10]. The multiplication operations during solving *G*_*d*_ can be implemented by using the CUBLAS library.

Furthermore, it can be found that *G*_*d*_ for each detector is irrelevant and can be parallel computing. However, for different time nodes, *G*_*d*_ cannot be calculated simultaneously because the calculation of the (*i* + 1)th time node of *G*_*d*_ depends on the *i*th time node of *G*_*d*_. Therefore, we can calculate *G*_*d*_ for all of the detectors for one time node at a time. At last, the structure of the whole *G*_*d*_ should be changed in order to solve the weight matrix conveniently.

#### 2.2.3. Weight Matrix Acceleration

As mentioned in (4), to solve the weight matrix, time convolution of several matrices should be calculated. Because the number of source-detector (sd for short) pairs is large and the size of *G*_*s*_ or *G*_*d*_ for each point and each time node is large, the whole procedure of solving the weight matrix is time consuming.

**Figure 2:**
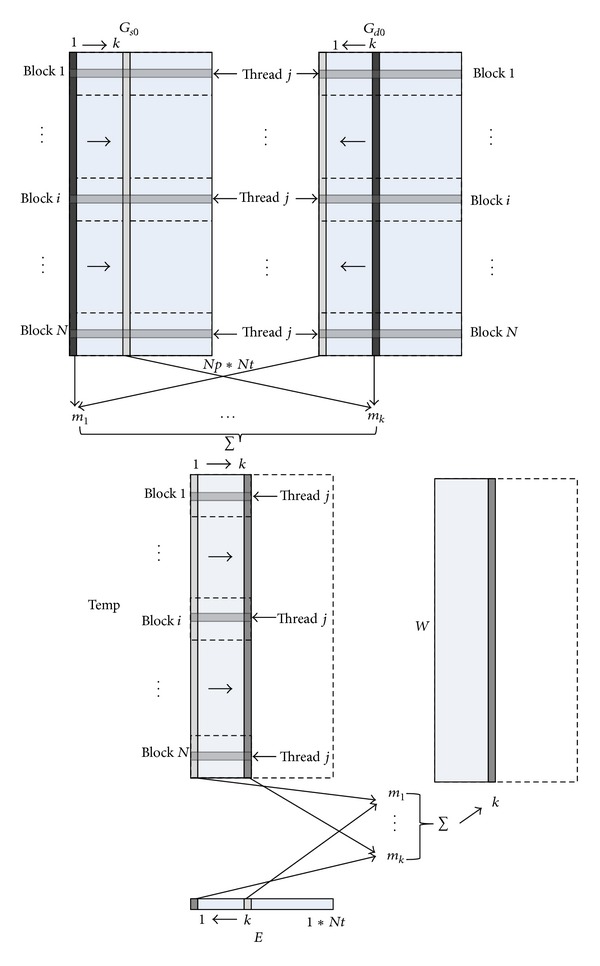
Principle of solving the weight matrix.

 CUDA language is adopted for the acceleration algorithm of solving the weight matrix.  Figure 2 shows the principle of the acceleration algorithm. *G*_*s*0_ and *G*_*d*0_ is Green's function for one source or detector for all the time nodes. The row stands for different mesh nodes and the column stands for different time nodes. It can be found that data of each row is irrelevant and only time convolution is calculated. Thus, data of each row can be distributed into different threads; therefore they can be implemented simultaneously. In this paper, the number of threads contained in each block is configured 256. The total block number is configured according to the row number of the matrix. Texture memory is used to load the matrix of *G*_*s*0_, *G*_*d*0_, and *E* because it can accelerate the data visiting speed with its cache. 

#### 2.2.4. NVMEX Technology

As the execution efficiency of Matlab is lower than C or Fortran, the time consuming subroutines are always programmed with C or Fortran and complied into binary MEX-files, which can be loaded and executed by the Matlab interpreter. 

However, subroutines with CUDA languages cannot be complied into MEX-files directly because CUDA language could not be recognized by the conventional compilers based on C or Fortran. Instead, this problem can be solved by the NVMEX technology, in which “NV” stands for NVIDIA. NVMEX technology connects Matlab and CUDA language conveniently and efficiently. With NVMEX technology, the codes based on CUDA are compiled into MEX-files by the “nvcc” complier and then called by the Matlab interpreter.

### 2.3. Experimental Setup

**Figure 3:**
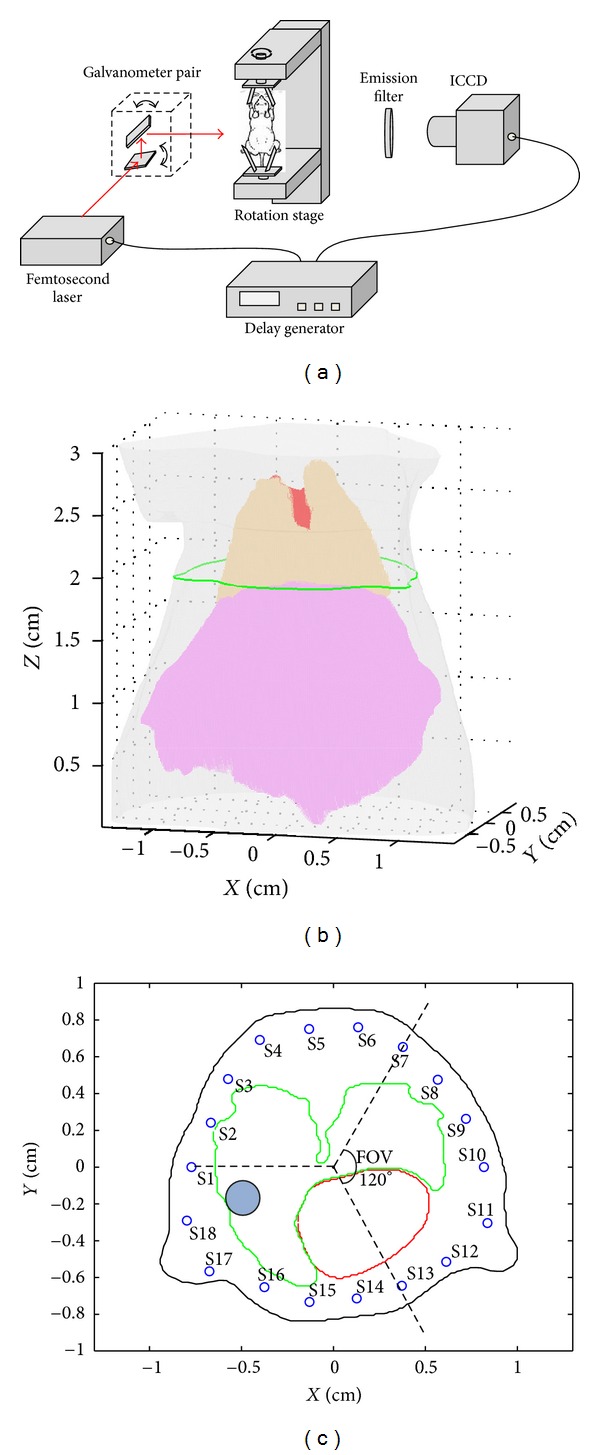
(a) Schematic of the free-space time-gated fluorescence tomography system. (b) The chest region of the digital mouse used for simulation. Different colors correspond to different tissue types (red: heart, orange: lungs, pink: liver, gray: adipose tissue). (c) Cross section of the digital mouse at the height of light source (green curve in (b)). The position of excitation lights and the field of view (FOV) with respect to source S1 are shown. The blue circle indicates the location of the fluorescent target.

Numerical experiments are performed to validate the performance of the acceleration strategy. The synthetic measurements are generated based on a free-space, time-gated FMT system, schematically depicted in Figure 3(a). The excitation light is an ultrafast laser emitting approximately 1ps pulses. The imaged mouse is suspended on a rotation stage and the laser beam is coupled to the surface of the mouse by a pair of galvanometer-controlled mirrors. At last, the transmitted light is detected by a high-speed intensified CCD (ICCD) at the opposite side of the excitation light [7].

In this simulation study, a 3D mouse atlas is employed which provides not only the complex surface but also the anatomical information [14]. We perform the numerical simulations based on the mouse chest region, so only the mouse torso from the neck to the bottom of the liver, as shown in Figure 3(b), is selected, with a height of 3 cm.

In the simulations, the mouse is suspended on the rotation stage and the rotation axis is defined as the *z*-axis. The mouse is rotated over 360° with 60° increments and the data collected consisted of 6 projections. The projection number is 6 because the computational size of the whole program will enlarge as the projection number increases and the memory consumption will exceed the limit of the computer. As shown in Figure 3(c), the field of view (FOV) of the detection with respect to each excitation source is 120°. A cylindrical fluorescent target with the height of 0.2 cm and radius of 0.1 cm is located at the (−0.31, −0.02, 1.93), which is indicated by the blue circle in Figure 3(c).

The simulations are performed in a heterogeneous mouse model. The absorption coefficient *μ*_*a*_ and the reduced scattering coefficient *μ*_*s*_′ shown in Table 1, which are calculated based on [15], are assigned to heart, lung, and liver to simulate photons propagation in biological tissues.

In order to evaluate the acceleration performance of the acceleration strategy, 6 simulated cases were performed. Configurations of these cases were the same except that the numbers of discretized mesh nodes and detectors were different. The excitation and emission intensity on the surface of the mouse model were calculated by a forward simulated program in advance.

For different cases, the reconstructed fluorescent distributions at the time node of 300 ps were shown as the early-photon results. For the reconstruction, the relaxation parameter of ART was *λ* = 0.1 and the number of iteration steps was 100.

At last, the programs are performed on an Intel(R) Core (TM) i7-2600 CPU (3.4 GHz) platform with 16 GB memory. A NVDIA Geforce GTX 460 graphics card with 336 cores is used for the acceleration strategy. The version number for the CUDA is 4.0. The contrasted programs are performed by Matlab 2008 and COMSOL Multiphysics 3.5 (COMSOL Inc, Stockholm, Sweden).

## 3. Results

### 3.1. The Necessity of the GPU Acceleration

For the simulated cases, the time consumption of each module by Matlab is shown in Table 2. The whole program is divided into 6 modules, among which the T4 and T5 modules are suitable for GPU-enabled acceleration. (In fact, the T3 module is also matrix operation. However, the time consumed by T3 is so short compared with the whole program that it is unnecessary to be accelerated.) 

**Figure 4:**
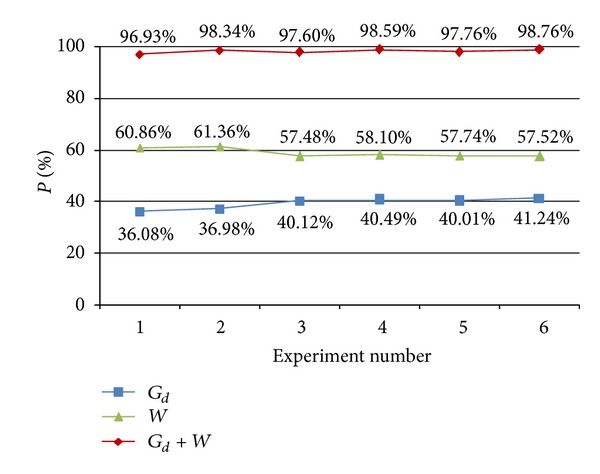
*P* values of the *G*_*d*_ (T4) module, *W* (T5) module and the *G*_*d*_ + *W* (T4 + T5) module.

It can be found that T4 and T5 modules are time consuming compared with other modules. In order to study the time occupancy quantitatively, we define *P* as the time percentage of each module to the total time for each case. The *P* values of the *G*_*d*_ (T4) module, *W* (T5) module, and *G*_*d*_ + *W* (T4 + T5) module are shown in Figure 4. It can be found that, for each case, the *P* value for the *G*_*d*_ + *W* module is more than 95%. As a result, we can reach the conclusion that the GPU acceleration is necessary.

### 3.2. Speedup Performance of the Acceleration Algorithms

For the 6 simulated cases, the fluorescent target is reconstructed by the Matlab program and the GPU acceleration strategy, respectively. Time consumption of each module by the two methods is recorded. Then the speedup ratios of the *G*_*d*_ acceleration algorithm, the weight matrix acceleration algorithm, and the whole acceleration strategy are studied, respectively. 

The time consumptions of *G*_*d*_ by both methods are showcased in Table 3. The speedup ratios of the *G*_*d*_-accelerating algorithm for different cases are shown in Table 3. It can be found that the speedup ratios decrease as the number of mesh nodes increases. The main reason is that, in the acceleration algorithm, the *G*_*d*_ is calculated for all the detection points and for one time node at a time. Therefore, the structure of the *G*_*d*_ should be adjusted into another form in order to suit for the following weight matrix calculation. The memory need for this step is huge, and as the scale increases, the memory can exceed the physical memory of the computer which leads to more time consumption.

The time consumptions of the weight matrix by both methods and the speedup ratios are showcased in Table 4. The speedup ratio for the weight matrix acceleration is more than 25, which is higher than that of the *G*_*d*_ acceleration algorithm. The reason is that the convolution operation is highly parallel, which makes it more easily for the GPU to achieve significant acceleration.

The speedup effect of the whole strategy is shown in Table 5. The final acceleration effect is a compromise of the acceleration of the computation between *G*_*d*_ and *W*. 

### 3.3. Accuracy of the Acceleration Strategy

In the GPU acceleration strategy, arithmetic operations are performed with single precision, because the use of double-precision operations results in increased memory requirements and a reduction of speedup performance. However, operations with single precision may bring in some errors compared with the double-precision operations by the Matlab.

**Figure 5:**
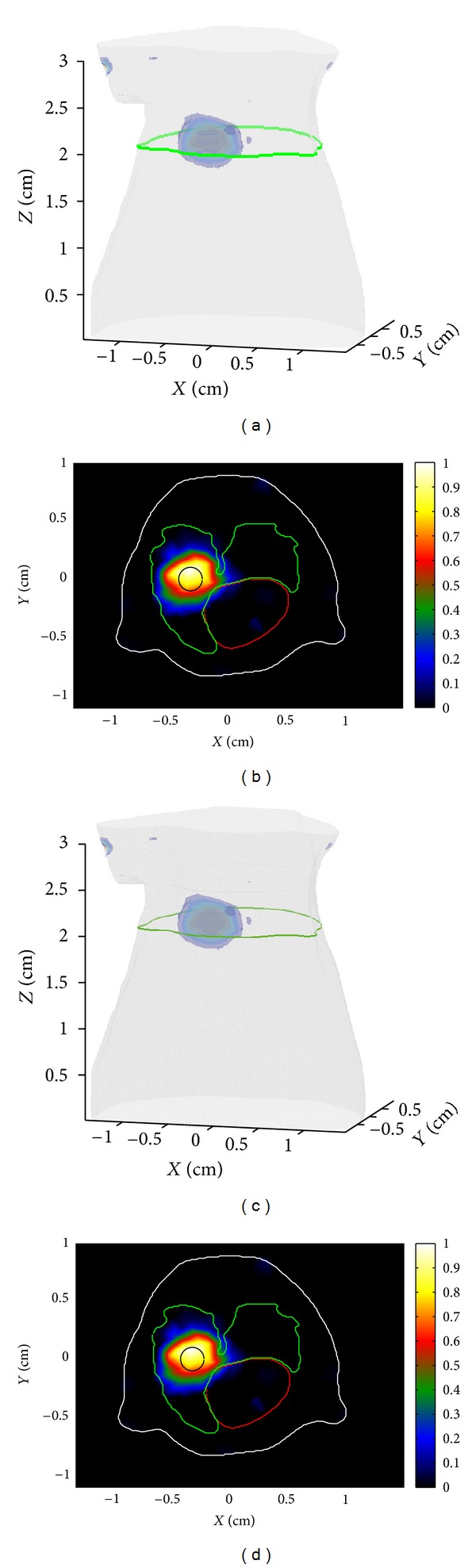
Reconstruction of the fluorescent target performed by Matlab and the GPU acceleration strategy. The first row shows the results reconstructed by Matlab while the second row shows the results reconstructed by the acceleration strategy. (a, c) The 3D views of the reconstructed results. (b, d) The cross-sections corresponding to the green curve lines in the 3D views. The black circles in (b, d) indicate the true locations of the fluorescent targets.

In order to study the error brought in by the single-precision operation in GPU. One simulated case with 4697 mesh nodes and 1409 detectors is selected to study the accuracy of reconstruction result (in fact, all the cases have the same conclusion and only one case is shown). Besides, 10% zero-mean, Gaussian noise was added to the synthetic data to simulate the actual case.  Figure 5 shows the reconstructed results by Matlab and the GPU acceleration strategy.

Then, the max error between the results is calculated as follows:

(10) $$\begin{array}{c} \max\left|\frac{\eta_{\text{Matlab}}-\eta_{\text{GPU}}}{\eta_{\text{Matlab}}}\right|\times 100\%=0.15\%, \end{array}$$

where *η*_Matlab_ and *η*_GPU_ stand for the reconstructed fluorescent signals in each node by Matlab and GPU acceleration strategy, respectively. It can be found that the max error is 0.15%, which is negligible.

## 4. Discussions

In this paper, we introduced an acceleration strategy for the early-photon fluorescence molecular tomography with GPU. Results of several numerical simulation cases validate the feasibility of this acceleration strategy.

With the acceleration strategy, the speedup ratio is about 10 for different cases. Compared with the other GPU-enabled acceleration algorithms [9, 10], the speedup ratio is not very great. There are mainly two reasons. First, the step to solve *G*_*d*_ is mainly matrix inversion operations, which is less suitable for parallel acceleration compared with the operations of matrix multiplication and matrix convolution. Besides, the time consumed by the structure conversion of *G*_*d*_ cannot be neglected while the computational scale is large. Second, the contrasted program is executed by Matlab and the functions for matrix operations in Matlab have been optimized.

The efficiency of the whole acceleration strategy is decided by two factors: the time percentage of the parallel modules to the whole program and the speedup efficiency of each acceleration algorithm. It can be found that the speedup ratio of the weight matrix algorithm is larger than that of *G*_*d*_. The cases studied in this paper are focused on different computational sizes and the projection number for each case is 6 for simplicity. If the projection number increases while the numbers of mesh nodes and detectors remain the same, the time percentage of the weight matrix module will increase. Therefore, for these cases, the final speedup ratio will be higher. 

For the *G*_*d*_ acceleration algorithm, the speedup ratio is not very remarkable. Future work will focus on improving its performance. In fact, the stuffing matrix produced by the FEM is a sparse matrix and the sparsity is used while the matrix inversion operations are performed in Matlab. However, the sparsity has not been utilized in the GPU acceleration algorithm. It is believed that the utilization of the sparsity of matrix will further improve speedup ratio of the *G*_*d*_ acceleration algorithm.

We performed several cases of different parameters to test the acceleration strategy. The imaging quality is improved when the numbers of mesh nodes and detectors increase. More detectors result in better spatial resolution and finer meshes will provide more details in the reconstructed results [16]. However, this paper is focused on the performance of the acceleration strategy for different simulation cases. The relationship between the experimental parameters and the reconstructed results is not the key point and is less considered.

In conclusion, we accelerated the early-photon fluorescence molecular tomography with GPU. Feasibility of this acceleration strategy was confirmed by several simulations. The accelerated results showed few errors while the time consumption was significantly reduced.

## Figures and Tables

**Table 1:** Optical parameters of different tissues of the digital mouse model.

Material	Heart	Lung	Liver	Background
μ_*a*_(cm^−1^)	0.156	0.516	0.935	0.1
μ_*s*_′ (cm^−1^)	9.0	21.2	6.4	10

**Table 2:** Time cost of each module in the Matlab program.

Experiment no.	Mesh nodes	Detectors	T1 (s) Load data	T2 (s) Assemble *K*	T3 (s) Form *G*_*s*_	T4 (s) Form *G*_*d*_	T5 (s) Form *W*	T6 (s) Solve *η*
1	3074	710	0.03	5.44	1.96	128.25	216.35	3.47
2	3074	1409	0.03	4.73	1.93	250.54	415.79	4.56
3	3881	710	0.03	5.03	3.13	195.13	279.57	3.48
4	3881	1409	0.03	5.46	3.20	386.06	553.90	4.78
5	4697	710	0.03	5.87	4.09	254.56	367.33	4.29
6	4697	1409	0.03	5.60	4.03	492.57	686.95	5.10

**Table 3:** Time comparisons of forming *G*_*d*_ consumed by Matlab and CUDA.

Experiment no.	Mesh nodes	Detectors	Time (s) Matlab	Time (s) CUDA	Speedup ratio
1	3074	710	128.25	19.89	6.4
2	3074	1409	250.54	25.08	10.0
3	3881	710	195.13	36.00	5.4
4	3881	1409	386.06	43.91	8.8
5	4697	710	254.56	55.88	4.6
6	4697	1409	492.57	89.15	5.5

**Table 4:** Time comparisons of forming the weight matrix consumed by Matlab and CUDA.

Experiment no.	Mesh nodes	Detectors	Time (s) Matlab	Time (s) CUDA	Speedup ratio
1	3074	710	216.35	8.46	25.6
2	3074	1409	415.79	16.72	24.9
3	3881	710	279.57	9.70	28.8
4	3881	1409	553.9	19.78	28.0
5	4697	710	367.33	10.65	34.5
6	4697	1409	686.95	21.53	31.9

**Table 5:** Time comparisons of the whole strategy consumed by Matlab and CUDA.

Experiment no.	Mesh nodes	Detectors	Time (s) Matlab	Time (s) CUDA	Speedup ratio
1	3074	710	355.50	39.23	9.1
2	3074	1409	677.58	52.33	12.9
3	3881	710	486.37	57.53	8.5
4	3881	1409	953.43	76.51	12.5
5	4697	710	636.17	80.83	7.9
6	4697	1409	1194.28	125.77	9.5
